# Overexpression of Bcl2 in Osteoblasts Inhibits Osteoblast Differentiation and Induces Osteocyte Apoptosis

**DOI:** 10.1371/journal.pone.0027487

**Published:** 2011-11-17

**Authors:** Takeshi Moriishi, Zenjiro Maruyama, Ryo Fukuyama, Masako Ito, Toshihiro Miyazaki, Hideki Kitaura, Hidetake Ohnishi, Tatsuya Furuichi, Yosuke Kawai, Ritsuko Masuyama, Hisato Komori, Kenji Takada, Hiroshi Kawaguchi, Toshihisa Komori

**Affiliations:** 1 Department of Cell Biology, Graduate School of Biomedical Sciences, Nagasaki University, Nagasaki, Japan; 2 Department of Sensory and Motor System Medicine, University of Tokyo, Tokyo, Japan; 3 Laboratory of Pharmacology, Hiroshima International University, Kure, Japan; 4 Department of Radiology and Radiation Biology, Graduate School of Biomedical Sciences, Nagasaki University, Nagasaki, Japan; 5 Division of Orthodontic and Biomedical Engineering, Graduate School of Biomedical Sciences, Nagasaki University, Nagasaki, Japan; 6 Department of Orthodontics and Dentofacial Orthopedics, Faculty of Dentistry, Osaka University, Osaka, Japan; 7 Department of Regenerative Oral Surgery, Graduate School of Biomedical Sciences, Nagasaki University, Nagasaki, Japan; 8 Division of Orthodontics and Dentofacial Orthopedics, Graduate School of Dentistry, Tohoku University, Sendai, Japan; 9 Laboratory Animal Facility, Research Center for Medical Sciences, School of Medicine, Jikei University, Tokyo, Japan; Instituto Nacional de Câncer, Brazil

## Abstract

Bcl2 subfamily proteins, including Bcl2 and Bcl-X_L_, inhibit apoptosis. As osteoblast apoptosis is in part responsible for osteoporosis in sex steroid deficiency, glucocorticoid excess, and aging, bone loss might be inhibited by the upregulation of Bcl2; however, the effects of Bcl2 overexpression on osteoblast differentiation and bone development and maintenance have not been fully investigated. To investigate these issues, we established two lines of osteoblast-specific *BCL2* transgenic mice. In *BCL2* transgenic mice, bone volume was increased at 6 weeks of age but not at 10 weeks of age compared with wild-type mice. The numbers of osteoblasts and osteocytes increased, but osteoid thickness and the bone formation rate were reduced in *BCL2* transgenic mice with high expression at 10 weeks of age. The number of BrdU-positive cells was increased but that of TUNEL-positive cells was unaltered at 2 and 6 weeks of age. Osteoblast differentiation was inhibited, as shown by reduced *Col1a1* and *osteocalcin* expression. Osteoblast differentiation of calvarial cells from *BCL2* transgenic mice also fell in vitro. Overexpression of *BCL2* in primary osteoblasts had no effect on osteoclastogenesis in co-culture with bone marrow cells. Unexpectedly, overexpression of *BCL2* in osteoblasts eventually caused osteocyte apoptosis. Osteocytes, which had a reduced number of processes, gradually died with apoptotic structural alterations and the expression of apoptosis-related molecules, and dead osteocytes accumulated in cortical bone. These findings indicate that overexpression of *BCL2* in osteoblasts inhibits osteoblast differentiation, reduces osteocyte processes, and causes osteocyte apoptosis.

## Introduction

Bone is a dynamic tissue that is constantly undergoing remodeling by osteoblasts and osteoclasts, and bone volume is determined by the differentiation and function of osteoblasts and osteoclasts. Osteoblasts, which differentiate from multipotent mesenchymal cells, express bone matrix protein genes at different levels depending on the maturity of the cells. Mesenchymal cells and preosteoblasts weakly express *Col1a1*, but osteoblasts show increased levels. Immature osteoblasts express *osteopontin*, and mature osteoblasts strongly express *osteocalcin*. Mature osteoblasts are finally embedded in the bone matrix to become osteocytes. Osteocytes located in lacunae establish an extensive intracellular and extracellular communication system via gap junction-coupled cell processes and canaliculi, through which cell processes pass throughout bone, and the communication system is extended to osteoblasts on the bone surface [1], [2]. The lacunocanalicular network formed by osteocytes is thought to be an ideal mechanosensory system and suitable for mechanotransduction, by which mechanical energy is converted into electrical and/or biochemical signals [3], [4], [5], [6], [7], [8].

Osteoblast apoptosis plays an important role in bone development and maintenance. It is estimated that 60–80% of osteoblasts that originally assembled at the resorption pit die by apoptosis. Further, bone loss caused by sex steroid deficiency, glucocorticoid excess, or aging is caused in part by osteoblast apoptosis, and PTH, bisphosphonate, and calcitonin exert anabolic action on bone by inhibiting osteoblast apoptosis [9], [10], [11], [12], [13], [14], [15], [16]. Bcl2 subfamily proteins, including Bcl2 and Bcl-X_L_, inhibit apoptosis through prevention of the release of caspase activators from mitochondria by inhibiting Bax subfamily proteins [17]. Thus, bone loss caused by sex steroid deficiency, glucocorticoid excess, or aging might be inhibited by the upregulation of Bcl2; however, the effects of overexpression of Bcl2 in osteoblast differentiation and bone development and maintenance have not been fully investigated.

Osteocyte apoptosis is a relatively common event in both pathological and healthy human bone and osteocyte apoptosis caused by microdamage precedes intracortical remodeling, raising the possibility that the apoptotic process might generate a signal used in targeted osteoclastic bone resorption [8]. Estrogen withdrawal and glucocorticoids induce osteocyte apoptosis, and estrogen and bisphosphonates attenuate osteocyte apoptosis [16], [18], [19]. The message transmitted by osteocyte apoptosis is considered to travel through the canalicular network to the surface of bone tissue and to be sent on to progenitor cells, leading to the initiation signal for remodeling, thereby stimulating the bone resorption/formation cycle [20]. Distinct from osteoblasts, osteocyte apoptosis represents cumulative death because cellular debris cannot be removed by phagocytes until the surrounding bone is resorbed, and TUNEL reactivity is retained in osteocyte lacunae long after osteocyte death [21].

To evaluate the effects of the overexpression of *BCL2* in osteoblasts, we examined osteoblast-specific *BCL2* transgenic mice. Here, we show that overexpression of *BCL2* in osteoblasts inhibited osteoblast differentiation, reduced osteocyte processes, and caused osteocyte apoptosis.

## Materials and Methods

### Ethics statement

Prior to the study, all experiments were reviewed and approved by the Animal Care and Use Committee of Nagasaki University Graduate School of Biomedical Sciences. (Permit Number: 0906170767-4).

### Mice

To generate transgenic mice with osteoblasts that express human *BCL2*, *BCL2* cDNA was inserted into the mammalian expression vector pNASSβ (CLONTECH, Shiga, Japan) by replacing the β-galactosidase gene at Not I sites, and the 2.3 kb osteoblast-specific promoter region of mouse *Col1a1* [22] was inserted into pNASSβ at EcoRI-XhoI sites. The 2.3 kb *Col1a1* promoter was a kind gift from B. de Crombrugghe (University of Texas M. D. Anderson Cancer Center). Transgenic mice were generated as previously described [23], and transgenic lines were maintained against a B6C3H F_1_ background. Until 2 weeks of age, both genders were used for analyses, but only male mice were used for analyses after 4 weeks of age. Tg(H) were mated with *p53* ^+/−^ mice [24], and *p53* ^−/−^tg(H) was finally generated. *p53* ^+/−^ mice were a kind gift from M. Katsuki (National Institute for Basic Biology). Prior to the study, all experiments were reviewed and approved by the Animal Care and Use Committee of Nagasaki University Graduate School of Biomedical Sciences.

### Histological analysis

Bone histomorphometric analyses were performed as previously described [23]. For assessment of dynamic histomorphometric indices, mice were injected with calcein 6 d and 2 d before sacrifice at a dose of 0.16 mg/10 g body weight. For histological analyses of the long bones, mice were sacrificed and fixed in 4% paraformaldehyde/0.01 M phosphate-buffered saline, and the long bones were decalcified in 10% EDTA (pH 7.4) and embedded in paraffin. Sections (3–7 µm thick) were stained with hematoxylin and eosin (H-E) or stained for TUNEL using the ApopTag® system (Intergen, Burlington, MA), or subjected to immunohistochemistry using monoclonal anti-human BCL2 antibody (Abcam, Cambridge, UK). For the BrdU incorporation study, mice of 2 and 6 weeks of age were injected intraperitoneally with 100 µg BrdU/g body weight and sacrificed 1 hour later. Sections were stained with the BrdU staining kit (Zymed, San Francisco, CA). In the counting of TUNEL-positive or BrdU-positive osteoblastic cells, only cells in the distal primary spongiosa of femurs, which were recognized as osteoblastic cells from their morphology and attachment to the trabecular bone, were counted. Bone canalicular staining (silver impregnation staining) was performed according to the method previously described [25]. Ultrastructural analysis was performed using a transmission electron microscope (H-7100; Hitachi, Tokyo, Japan) as previously described [26]. To observe the three-dimensional ultrastructure of osteocytes, the HCl-collagenase method [27] was applied. The treated specimens were observed under a scanning electron microscope (S-3500N; Hitachi).

### Real-time RT-PCR and Western blot analyses

Muscle, connective tissue, and periosteum were removed from femurs and tibiae, and the bones were cut at the metaphyses. After hematopoietic cells in the diaphyses of femurs and tibiae were flushed out with PBS, osteoblast-enriched cells were collected using a micro-intertooth brush (Kobayashi Pharmaceutical Co. Ltd. Osaka, Japan). The remaining bone was used as a source of osteocyte-enriched cells. Nearly complete removal of osteoblasts from the endosteum by the micro-intertooth brush was confirmed using a scanning electron microscope (Miniscope TM-1000; Hitachi). Total RNA was extracted using ISOGEN (Wako, Osaka, Japan), and real-time RT-PCR was performed as previously described [28]. Primer sequences are shown (Table S1). We normalized the values to those of *Gapdh*. Using osteoblast-enriched or osteocyte-enriched whole-cell lysates, Western blot analysis was performed using the following antibodies: anti-BCL2, anti-p53, anti-Cul1, anti-Bim, anti-Cyclin D1, anti-t-Bid, anti-Bad, and anti-Bcl-X_L_ (BD); anti-Bax, anti-p21, anti-p27, anti-p16, anti-actin (Santa Cruz Biotechnology, Santa Cruz, CA); anti-p57 (Sigma, St. Louis, MO); anti-cleaved caspase-3 (Chemicon, Temecula, CA); anti-Hif-1α (Zymed, San Francisco, CA); and anti-Mcl1 (Abcam).

### Micro-CT analysis

Quantitative micro-CT analysis was performed with a micro-CT system (μCT-20; Scanco Medical, Brüttisellen, Switzerland). Data from scanned slices were used for three-dimensional analysis to calculate femoral morphometric parameters. Trabecular bone parameters were measured on a distal femoral metaphysis. Approximately 2.4 mm (0.5 mm from the growth plate) were cranio-caudally scanned and 200 slices in 12 µm increments were made.

### Cell culture experiments

Primary osteoblasts were isolated from newborn calvaria by sequential digestion with 0.1% collagenase A and 0.2% dispase. Osteoblastic cells from the third to fifth fraction were pooled and used for MTT assay and osteoblast differentiation. To examine osteoblast differentiation, staining for alkaline phosphatase (ALP) activity and mineralization was performed as previously described [29]. Mineralization was quantified using VHX-1000 (KEYENCE) and Image J. Primary calvarial cells derived from C57BL/6 embryos at embryonic day 18.5 were also prepared by culturing small pieces of calvaria in three-dimensional collagen gel for 10–14 days as described previously [29], and used for the analyses of osteoblast differentiation, apoptosis, and osteoclastogenesis. To examine apoptosis, calvarial cells were infected with EGFP-expressing retrovirus or human *BCL2*- and EGFP-expressing retrovirus, which was generated by a bicistronic expression vector (human *BCL2* internal ribosome entry site (IRES)-EGFP), plated on 8-well chamber slides (Nalge Nunc, Rochester, NY) at a density of 1×10^4^/well, and treated with PBS or 1 mM hydrogen peroxide for 3 hrs after confluence. TUNEL-positive cells were detected using the ApopTag® system (Intergen, Burlington, MA). The co-culture of primary osteoblasts and bone marrow cells was performed as previously described [26]. In all of the retrovirus infection experiments, the amount of retrovirus was adjusted to obtain similar EGFP signals in the EGFP-expressing retrovirus and human *BCLl2*- and EGFP-expressing retrovirus infections.

### Statistical analysis

Statistical analyses were performed using Student's *t*-test. P<0.05 was considered significant.

## Results

### Bone mass increased in *Bcl2* transgenic mice at 6 weeks but not 10 weeks of age

**Figure 1:**
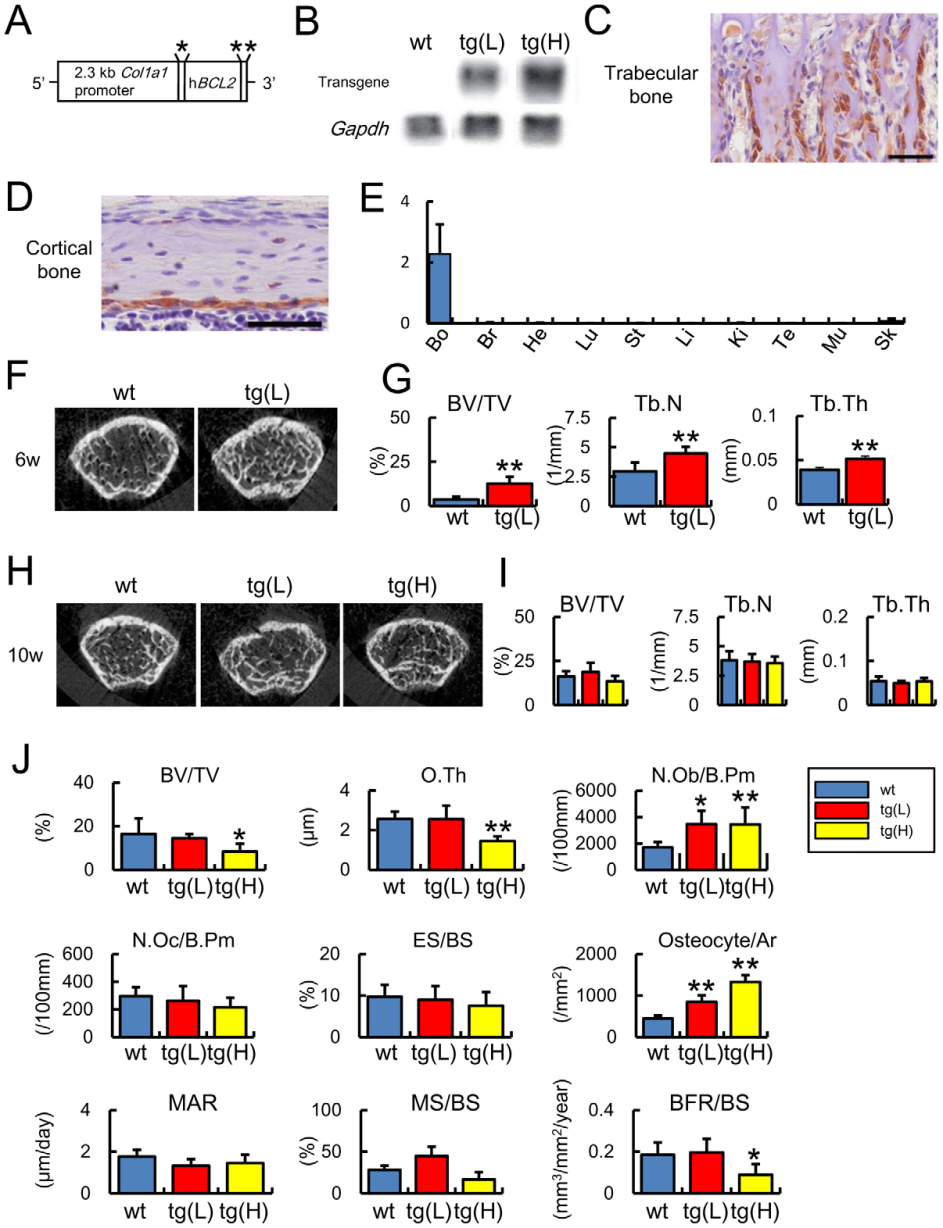
Generation of *BCL2* transgenic mice and micro-CT and bone histomorphometric analyses. (A) Diagram of the DNA construct used to generate transgenic mice that express human *BCL2* under the control of the *Col1a1* promoter. *Intron from SV40 containing splice donor and acceptor sites, **polyadenylation signal from SV40. (B) Northern blot analysis of the transgene. The expression level of the transgene was examined by Northern blot analysis using total RNA that had been extracted from the femurs of two *BCL2* transgenic mouse lines (tg(L) and tg(H)) and wild-type mice (wt) at 5 weeks of age. Twenty micrograms of RNA were loaded and *Gapdh* was used as an internal control. (C, D) Immunohistochemical analysis using anti-human BCL2 antibody. Sections of trabecular bone (C) and cortical bone (D) from tg(L) at 2 weeks of age were reacted with anti-human BCL2 antibody, which does not react with mouse Bcl2. Scale bars = 50 µm. (E) Real-time RT-PCR analysis of the transgene expression. RNA was extracted from bone (Bo), brain (Br), heart (He), lung (Lu), stomach (St), liver (Li), kidney (Ki), testis (Te), muscle (Mu), and skin (Sk) of tg(L) at 10 weeks of age. (F–I) Micro-CT analysis. F and H, Two-dimensional axial image of distal femoral metaphysis of male mice at 6 weeks of age (F) and 10 weeks of age (H). G and I, Trabecular bone volume (BV/TV), trabecular number (Tb.N), and trabecular thickness (Tb.Th) at 6 weeks of age (G) and 10 weeks of age (I) are shown. Trabecular bone parameters were measured on distal femoral metaphysis. Data are presented as the mean ± S.D. of 4–18 male mice. (J) Bone histomorphometric analysis. The trabecular bone volume (bone volume/tissue volume, BV/TV), osteoid thickness (O.Th), number of osteoblasts (N.Ob/B.Pm), number of osteoclasts (N.Oc/B.Pm), eroded surface (ES/BS), number of osteocytes (N.Ot/Ar), mineral apposition rate (MAR), mineralizing surface (MS/BS), and bone formation rate (BFR/BS) were compared among male wild-type mice (blue), tg(L) (red), and tg(H) (yellow) at 10 weeks of age. Data are presented as the mean ± S.D. of 5–10 mice. *vs. wild-type mice. *P<0.05, **P<0.01. B.Pm, bone perimeter; BS, bone surface.

We established two lines of human *BCL2* transgenic mice under the control of mouse 2.3 kb *Col1a1* promoter with different expression levels, a transgenic line with low expression of *BCL2* (tg(L)) and a transgenic line with high expression of *BCL2* (tg(H)) (Fig. 1A, B). Transgene expression was examined by immunohistochemistry at 2 weeks of age, and was specifically detected in osteoblasts and young osteocytes (Fig. 1C, D). We also examined the expression of the transgene in other tissues by real-time RT-PCR. Although the transgene was weakly detected in the skin as previously described [22], it was barely detectable in the brain, heart, lung, stomach, liver, kidney, testis, and muscle (Fig. 1E). We examined the bone phenotypes at a growing stage (6 weeks of age) and an adult stage (10 weeks of age). On micro-computed tomography (micro-CT) analysis at 6 weeks of age, bone volume, trabecular number, and trabecular thickness were increased in tg(L) compared with wild-type mice (Fig. 1F, G). Bone histomorphometric analysis showed that the osteoblast surface was markedly increased in tg(L) compared with wild-type mice at 6 weeks of age (osteoblast surface: wild-type mice; 33.9±9.2%, tg(L); *60.4±5.3%, n = 6–8, *p<0.01); however, the parameters of trabecular bone in tg(L) and tg(H) were comparable to those in wild-type mice at 10 weeks of age on micro-CT analysis (Fig. 1H, I). At 10 weeks of age, peripheral quantitative computed tomography (pQCT) analysis showed that the mineral density of cortical bone was reduced in tg(H) but not in tg(L) compared with that in wild-type mice (Figure S1). Bone histomorphometric analysis at 10 weeks of age showed that the numbers of osteoblasts and osteocytes were increased in both tg(L) and tg(H) compared with those in wild-type mice, but that the bone volume, osteoid thickness, and bone formation rate were not increased in tg(L) and reduced in tg(H) compared with those in wild-type mice, irrespective of the similar levels of osteoclast parameters among wild-type mice, tg(L), and tg(H) (Fig. 1J). The phenotypic differences observed in *BCL2* transgenic mice at 6 and 10 weeks of age were likely to be due to the change in transgene expression, because the level of transgene expression at 10 weeks of age was about half of that at 6 weeks of age in real-time RT-PCR analysis (data not shown).

### Osteoblast proliferation was increased without augmentation of apoptosis and osteoblast differentiation was impaired in *BCL2* transgenic mice

**Figure 2:**
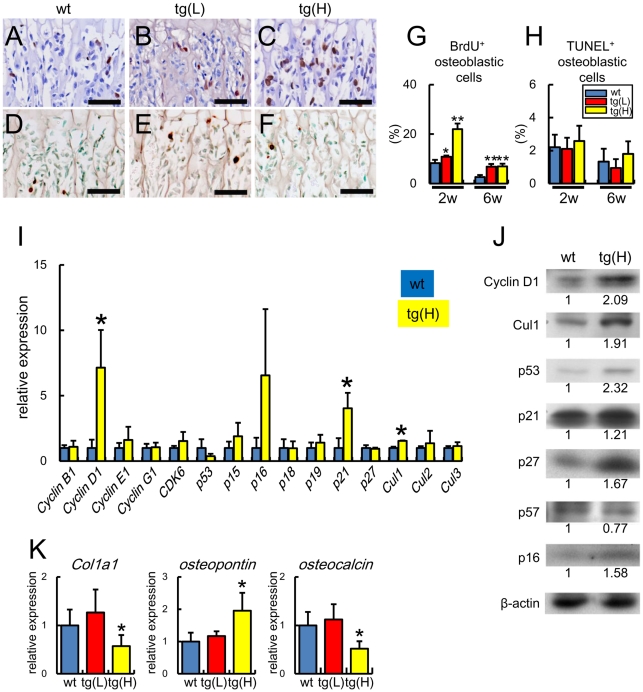
Analyses of osteoblast proliferation, apoptosis, and differentiation in *BCL2* transgenic mice. (A–H) BrdU labeling (A–C) and TUNEL staining (D–F) of sections of distal femoral metaphysis from wild-type mice (A, D), tg(L) (B, E), and tg(H) (C, F) at 2 weeks of age. The sections were counterstained with hematoxylin (A–C) and methyl green (D–F). Scale bars = 50 µm. BrdU-positive osteoblastic cells (G) and TUNEL-positive osteoblastic cells (H) of wild-type mice (blue), tg(L) (red), and tg(H) (yellow) were counted and shown as a percentage of the number of osteoblastic cells. Data are presented as the mean ± S.D. of 4–12 mice. *vs. wild-type mice. *P<0.05, **P<0.01. (I) Real-time RT-PCR analysis. RNA was extracted from the osteoblast-enriched fraction of femurs and tibiae of male wild-type mice (blue) and tg(H) (yellow) at 6 weeks of age and the expression of cell cycle-related genes was examined. The values of wild-type mice were defined as 1, and relative levels are shown. Data are presented as the mean ± S.D. of 5–8 mice. *vs. wild-type mice. *P<0.05. (J) Western blot analysis. Proteins were extracted from the osteoblast-enriched fraction of femurs and tibiae of male wild-type mice (wt) and tg(H) at 6 weeks of age. The ratios of the intensities of the bands are shown against β-actin. Similar results were obtained in three independent experiments and representative data are shown. (K) Real-time RT-PCR analysis of *Col1a1*, *osteopontin*, and *osteocalcin*. RNA was extracted from femurs of wild-type mice (wt), tg(L), and tg(H) at 2 weeks of age. The values of wild-type mice were defined as 1, and relative levels are shown. Data are presented as the mean ± S.D. of 5 mice. *vs. wild-type mice. *P<0.05.

As the osteoblast density was increased in *BCL2* transgenic mice, we examined the effect of BCL2 on osteoblast proliferation by BrdU labeling. The percentage of BrdU-positive cells was increased mildly in tg(L) and markedly in tg(H) at 2 weeks of age and increased similarly in tg(L) and tg(H) at 6 weeks of age compared with that in wild-type mice at the respective age (Fig. 2A–C, G). We also examined osteoblast apoptosis by terminal deoxynucleotidyl transferase-mediated deoxyuridine triphosphate nick end labeling (TUNEL). Similar percentages of TUNEL-positive osteoblastic cells were observed among wild-type mice, tg(L), and tg(H) (Fig. 2D–F, H).

As osteoblast proliferation was increased in *BCL2* transgenic mice, we examined the expression of cell cycle-related molecules by real-time RT-PCR and Western blot analyses using osteoblast-enriched samples from femurs and tibiae (Fig. 2I, J). The mRNA and protein levels of cyclin D1, p21, and Cul1 were increased and the protein levels of p53, p27, and p16 were increased.

Next, we examined the expression of osteoblast differentiation markers. Real-time RT-PCR analysis showed similar expression levels of *Col1a1*, *osteopontin*, and *osteocalcin* in tg(L), but reductions in *Col1a1* and *osteocalcin* expression and an increase in *osteopontin* expression in tg(H) compared with the respective levels in wild-type mice at 2 weeks of age (Fig. 2K). Similar results were also obtained by Northern blot analysis at 4 weeks of age and in situ hybridization at 8 weeks of age (Figure S2). These findings indicate that osteoblast differentiation was impaired in tg(H). Since the osteoblast density but neither the bone volume nor bone formation rate were increased in tg(L) and tg(H) at 10 weeks of age (Fig. 1J), osteoblast function should have been impaired in both tg(L) and tg(H).

### Overexpression of *BCL2* in osteoblasts inhibits osteoblast differentiation and apoptosis but has no effect on osteoclastogenesis in vitro

**Figure 3:**
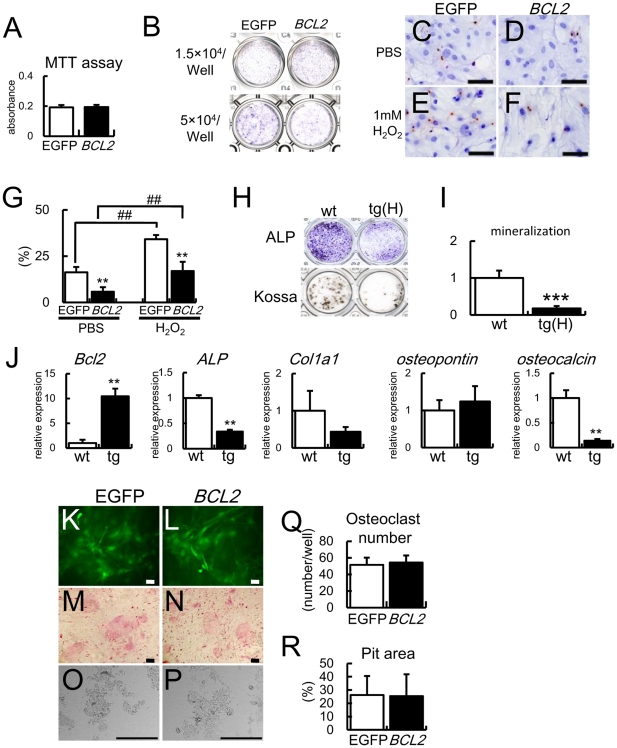
Analyses of osteoblast proliferation, apoptosis, and differentiation and osteoclastogenesis in vitro. (A) MTT assays. MTT assays were performed using wild-type primary osteoblasts infected with EGFP-expressing or *BCL2*-and-EGFP-expressing retrovirus. Data are presented as the mean ± S.D. of 16 wells. Similar results were obtained in two independent experiments and representative data are shown. (B) ALP activity. Primary osteoblasts prepared using three-dimensional collagen gel were infected with EGFP-expressing or *BCL2*- and EGFP-expressing retrovirus and seeded on 24-well plates at the indicated cell densities, and ALP activity was examined after culture for 6 days (1.5×10^4^ cells/well) or 4 days (5×10^4^ cells/well). Similar results were obtained in two independent experiments and representative data are shown. (C–G) Analysis of apoptosis in vitro. Primary osteoblasts were infected with EGFP-expressing (C, E) or *BCL2*- and EGFP-expressing (D, F) retrovirus. At confluence, the cells were treated with PBS (C, D) or 1 mM H_2_O_2_ (E, F) for 3 h and TUNEL staining was performed. The cells were counterstained with hematoxylin. Scale bars = 50 µm. The percentage of TUNEL-positive cells is shown as the mean ± S.D. of 5 wells (G). *vs. EGFP. **, ##P<0.01. Similar results were obtained in two independent experiments and representative data are shown. (H–J) Differentiation of primary osteoblasts from tg(H). Primary osteoblasts were prepared from newborn calvariae of wild-type mice and tg(H), seeded on 24 well plates at the density of 5×10^4^ cells/well, and ALP staining and von Kossa staining were performed after culture for 4 days and 15 days, respectively (H). I, Quantification of mineralization. RNA was extracted after culture for 4 days and real-time RT-PCR analysis was performed (J). The value of primary osteoblasts from wild-type mice was set as 1 and the relative levels are shown in I and J. Data are presented as the mean ± S.D. of 3 mice. *vs. wild-type mice, **p<0.01, ***p<0.001. Similar results were obtained in two independent experiments and representative data are shown. (K–R) Osteoclastogenesis in vitro. Primary osteoblasts were infected with retrovirus expressing either EGFP (K, M, O) or *BCL2*-EGFP (L, N, P), and cultured with bone marrow cells for 6 days. EGFP signal (K, L), TRAP staining (M, N), dentin slices (O, P), number of multinucleated TRAP-positive cells (Q), and Pit area (R) are shown. Scale bars = 20 µm (K, L); 50 µm (M, N); 200 µm (O, P). Similar results were obtained in two independent experiments and representative data are shown.

In contrast to enhanced osteoblast proliferation in *BCL2* transgenic mice, overexpression of *BCL2* had no effect on osteoblast proliferation in vitro (Figs. 2A–C, G, 3A). To examine the effect of Bcl2 on osteoblast differentiation, we first introduced *BCL2* into primary osteoblasts from wild-type mice by retrovirus and examined ALP activity. ALP activity in *BCL2*-introduced cells was increased compared with that in EGFP-introduced cells when the cells were seeded at 1.5×10^4^ cells/well, whereas it was reduced compared with that in EGFP-introduced cells when seeded at 5×10^4^ cells/well (Fig. 3B). To investigate why ALP activity was dependent on cell density, we examined the effect of BCL2 on apoptosis in vitro, because cell density is critical for osteoblast differentiation. Overexpression of *BCL2* in primary osteoblasts significantly reduced osteoblast apoptosis with or without hydrogen peroxide treatment (Fig. 3C–G). To minimize the effect of BCL2 on apoptosis during culture, primary osteoblasts from tg(H) were seeded at the high concentration (5×10^4^ cells/well). ALP activity, mineralization, and the expressions of *ALP* and *osteocalcin* were reduced compared with in wild-type mice (Fig. 3H–J).

To investigate the osteoblast function of supporting osteoclastogenesis, we co-cultured calvarial cells and bone marrow cells. Retroviral introduction of either EGFP or *BCL2* into primary osteoblasts showed similar numbers of tartrate-resistant acid phosphatase (TRAP)-positive cells and a similar resorption area in the co-culture (Fig. 3K–R).

### Osteocyte apoptosis in *BCL2* transgenic mice

**Figure 4:**
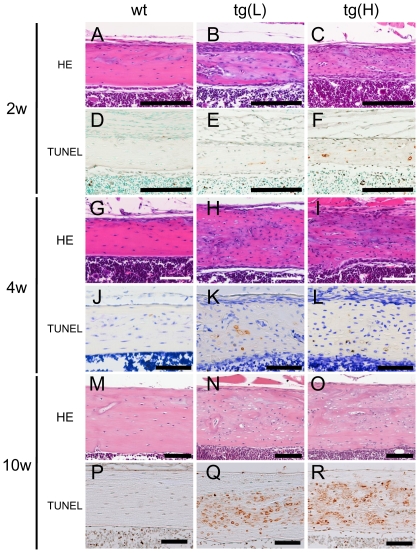
Osteocyte apoptosis in cortical bone. H-E (A–C, G–I, M–O) and TUNEL (D–F, J–L, P–R) staining of cortical bone at the diaphyses of femurs of wild-type mice (A, D, G, J, M, P), tg(L) (B, E, H, K, N, Q), and tg(H) (C, F, I, L, O, R) at 2 weeks (A–F), 4 weeks (G–L), and 10 weeks of age (M–R). Scale bars = 0.1 mm.

**Figure 5:**
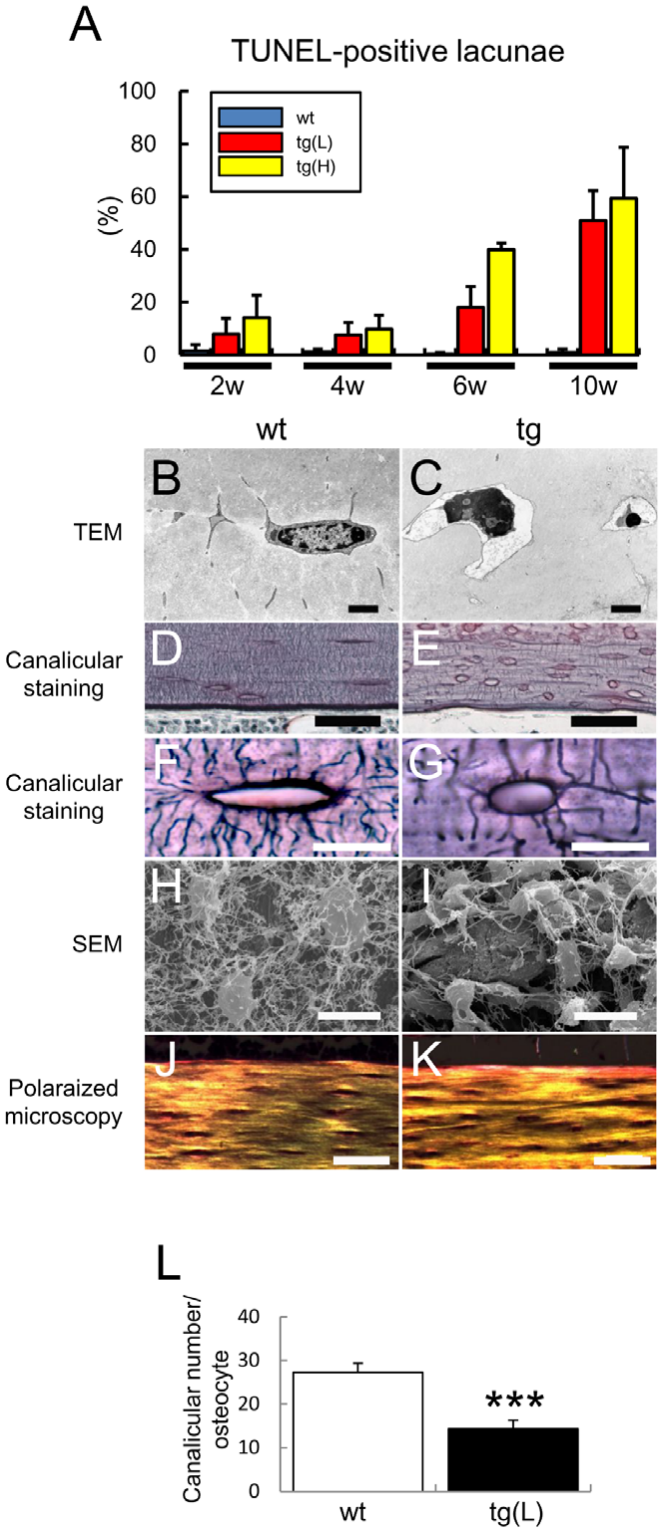
Histological analysis of osteocytes. (A) Frequency of TUNEL-positive lacunae. The number of TUNEL-positive lacunae was counted at 2 weeks, 4 weeks, 6 weeks, and 10 weeks of age, and presented as a percentage of the total number of lacunae in the cortical bone of femurs. (B, C) Images of osteocytes by transmission electron microscope (TEM) in the cortical bone of tibiae from male wild-type mouse (B) and tg(H) (C) at 10 weeks of age. Bars: 2 µm. (D–G) Canalicular staining of femurs from male wild-type mouse (D, F), tg(H) (E), and tg(L) (G) at 10 weeks of age. F and G show magnified views of osteocyte lacunae and canaliculi. Bars = 50 µm (D, E); 5 µm (F, G). (H, I) Images of young osteocytes by scanning electron microscope in the cortical bone of femurs from male wild-type mouse (H) and tg(H) (I) at 10 weeks of age. Bars: 10 µm. (J, K) Polarized microscopy of cortical bone at diaphyses of femurs in male wild-type mouse (J) and tg(L) (K) at 10 weeks of age. Bars: 50 µm. (L) Number of canaliculi. The number of canaliculi derived from each lacuna was counted. Four lacunae in the diaphysis of the femur were examined in each mouse. Data are presented as the mean ± S.D. of 7 male wild-type mice and 6 male tg(L) at 10 weeks of age. *vs. wild-type mice, ***p<0.001.

Surprisingly, empty lacunae or lacunae containing cell debris were increased in *BCL2* transgenic mice, and the increase was already evident in the distal femoral metaphysis of tg(H) at 1 week of age (Figure S3). To examine whether the osteocytes died by apoptosis, we performed TUNEL staining. About 1% of lacunae in the cortical bone at the diaphyses of femurs were TUNEL-positive in wild-type mice from 2 to 10 weeks of age, whereas the corresponding percentage was about 10% in both tg(L) and tg(H) at 2 and 4 weeks of age, 20% in tg(L) and 40% in tg(H) at 5–6 weeks of age, and 50% in tg(L) and 60% in tg(H) at 10 weeks of age (Figs. 4, 5A). After the death of osteocytes, the lacunae contained only cell debris but TUNEL reactivity was retained in the lacunae (Fig. 4), because the debris of dead osteocytes cannot be eliminated until the surrounding bone is resorbed. Thus, TUNEL-positive lacunae accumulated during bone development. To further confirm that the osteocytes in *BCL2* transgenic mice died by apoptosis, we observed them by transmission electron microscopy, which is the most reliable tool for evaluation of the type of cell death (Fig. 5B, C). Apoptotic structural alterations of osteocytes, such as cytoplasmic shrinkage, chromatin condensation, and nuclear disintegration, were observed in the lacunae of the cortical bone of tibiae of *BCL2* transgenic mice (Fig. 5C); however, we did not observe cellular swelling, disturbance of plasma membranes and membranes of cytoplasmic organelles, or swollen, electron-lucent nuclei, all of which are features of necrosis. Canaliculi and osteocyte processes were abundant and densely connected in wild-type mice, whereas the numbers of canaliculi and osteocyte processes were reduced depending on the expression levels of the transgene and were sparsely connected in *BCL2* transgenic mice (Fig. 5D–I, L, data not shown). Observation by polarized microscopy showed lamellar collagen deposition in the cortical bone of both wild-type mice and tg(L), although the collagen fibers were mildly disorganized in tg(L), probably due to the death of osteocytes (Fig. 5J, K).

### Expression of apoptosis-related genes in osteocytes and failure to rescue apoptosis by *p53* deletion in *BCL2* transgenic mice

**Figure 6:**
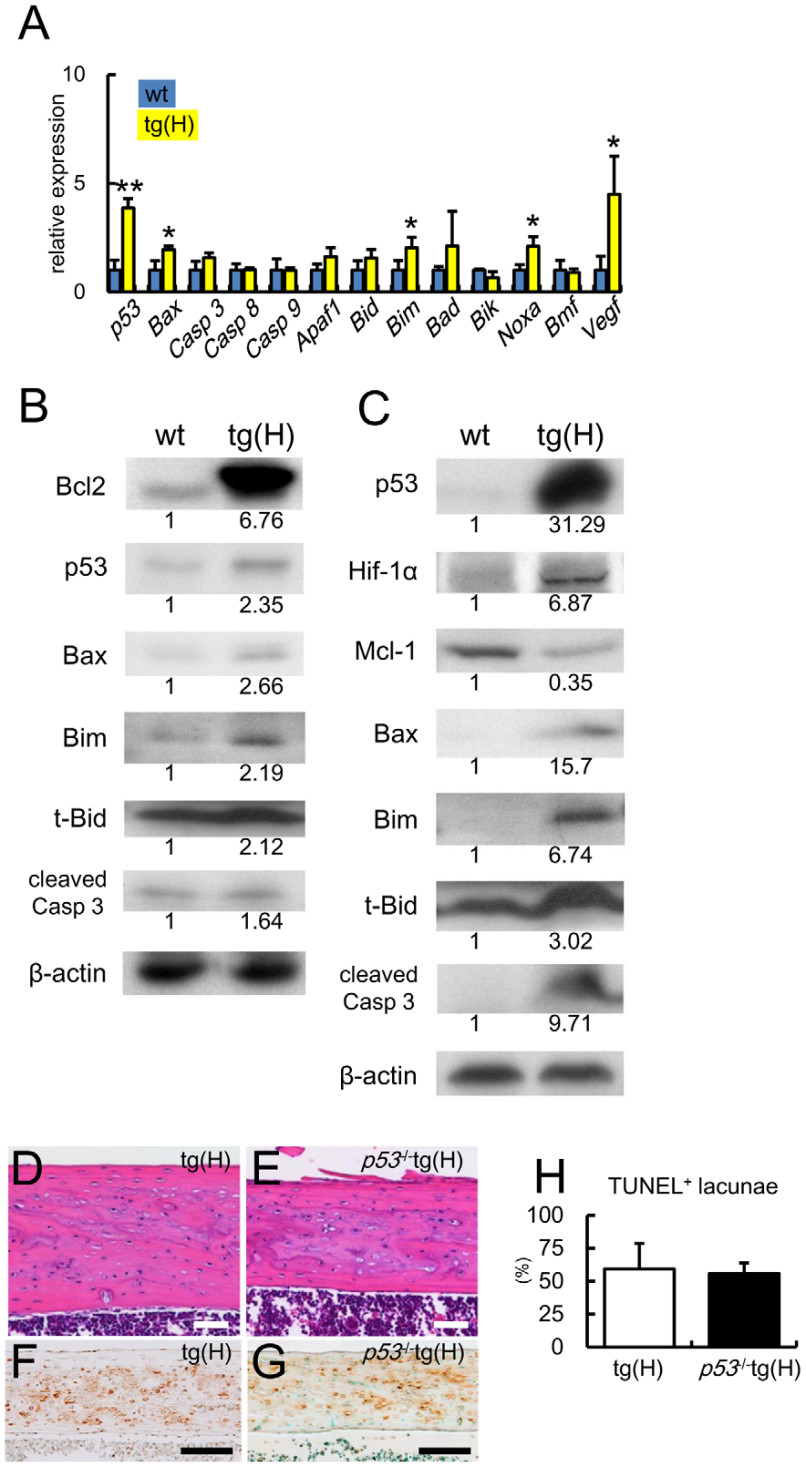
Expression of apoptosis-related molecules and TUNEL staining of cortical bone in *p53* ^−/−^tg(H). (A) Real-time RT-PCR analysis of apoptosis-related genes and *Vegf*. RNA was extracted from osteocyte-enriched samples of tibiae and femurs of male wild-type mice (blue) and tg(H) (yellow). The values of wild-type mice were defined as 1, and relative levels are shown. Data are presented as the mean ± S.D. of 4–10 mice at 5–6 weeks of age. *vs. wild-type mice. *P<0.05, **P<0.01. (B, C) Western blot analysis of apoptosis-related molecules using cell extract from osteoblast-enriched fraction (B) and osteocyte-enriched fraction (C) in male wild-type mice (wt) and tg(H) at 5–6 weeks of age. β-actin was used as an internal control. The anti-Bcl2 antibody used here reacts with both mouse and human Bcl2. Similar results were obtained in three independent experiments and representative data are shown. (D–H) Failure to rescue osteocyte apoptosis by the deletion of *p53*. H-E (D, E) and TUNEL (F, G) staining of sections of cortical bone at the diaphyses of femurs of male tg(H) (D, F) and *p53* ^−/−^tg(H) (E, G) and the frequencies of TUNEL-positive lacunae (H) at 10 weeks of age. Scale bars: 0.1 mm. Data are presented as the mean ± S.D. of 3 mice.

In accordance with the appearance of apoptotic cells, *p53*, *Bax*, *Bim*, *Noxa*, and *Vegf* were upregulated in the osteocyte-enriched samples from tg(H) compared with the respective levels in those from wild-type mice (Fig. 6A). On Western blot analyses using osteoblast-enriched samples, the protein levels of p53, Bax, Bim, and t-Bid in tg(H) were slightly increased and the protein level of cleaved caspase-3 in tg(H) was similar to the respective level in wild-type mice (Fig. 6B). On Western blot analyses using osteocyte-enriched samples, however, protein levels of p53, HIF-1α, Bax, Bim, t-Bid, and cleaved caspase-3 were apparently increased and that of Mcl1 was decreased in tg(H) compared with the respective level in wild-type mice (Fig. 6C), further supporting the histological observation that osteocytes died by apoptosis. As p53 expression was markedly increased in the osteocytes of *BCL2* transgenic mice, we mated tg(H) with *p53* ^+/−^ mice and finally generated *p53* ^−/−^tg(H) mice (Fig. 6D–G). The deletion of *p53* failed to inhibit osteocyte apoptosis in tg(H) (Fig. 6H).

## Discussion

We analyzed male *BCL2* transgenic mice, and osteoblast density was increased in tg(L) and tg(H) but the bone formation rate was similar or reduced in tg(L) and tg(H), respectively, compared with wild-type mice. Although the differentiation stage of osteoblasts was not examined in the previously reported *BCL2* transgenic mice, we found that osteoblast differentiation was inhibited in a manner dependent on transgene expression, as shown by the reduction in *Col1a1* and *osteocalcin* expression and the increase in *osteopontin* expression. Further, the inhibition of osteoblast differentiation was also observed in ex vivo experiments; however, the differentiation of osteoblasts derived from the previous *BCL2* transgenic mice was shown to be enhanced in vitro [30]. This controversial result may have been caused by an increase in the cell density of primary osteoblasts from *BCL2* transgenic mice compared with wild-type mice during culture, because the frequency of apoptosis in wild-type primary osteoblasts fell to one third by the introduction of *BCL2* (Fig. 3G) and the increased cell density accelerated osteoblast differentiation.

Although osteoblast proliferation was not examined in vivo in previously reported *BCL2* transgenic mice [31], we showed that osteoblast proliferation was enhanced in *BCL2* transgenic mice; however, introduction of *BCL2* failed to enhance the proliferation of wild-type primary osteoblasts (Fig. 3A), and primary osteoblasts from previous *BCL2* transgenic mice showed similar proliferation to those from wild-type mice [30]. Further, previous reports showed that Bcl2 inhibited cell proliferation by facilitating G0 arrest and delaying G0 to S phase transition in hematopoietic cells and fibroblasts [32], and various groups showed that p27 as well as p130 was elevated in *Bcl2*-overexpressing cells during arrest [33], [34], [35], [36], although overexpression of *Bcl2* in myocytes promoted proliferation [37]. Therefore, BCL2 may have no intrinsic ability to enhance osteoblast proliferation, and it is possible that the population of proliferating osteoblasts was increased by the deceleration of osteoblast differentiation in *BCL2* transgenic mice. The upregulation of cyclinD1 and Cul1, which is a component of an ubiquitin ligase complex targeting p21 and p27 [38], [39], in *BCL2* transgenic mice may reflect the accumulation of proliferating osteoblasts. Osteoblast apoptosis was not reduced in *BCL2* transgenic mice; however, osteoblast apoptosis should have been suppressed in *BCL2* transgenic mice because of an increase of proliferating osteoblasts, in which apoptosis would be increased by replication stress [40], and the suppression of apoptosis should have contributed to the increase in the number of proliferating osteoblasts.

A increase in osteocyte apoptosis was not observed in the previous *BCL2* transgenic mice [31]. The discrepancy of the osteocyte phenotype may be due to differences in the genetic background because the previous *BCL2* transgenic mice were CD-1 outbred mice. We observed an increase in osteocyte apoptosis not only in *BCL2* transgenic mice with a B6C3H F_1_ background but also in those with a C57BL/6 background, which were generated by backcrossing with C57BL/6 mice ten times (data not shown). Outbred mice may have been resistant to osteocyte apoptosis caused by overexpression of *BCL2* in osteoblasts. It is also possible that the expression level of the transgene in the previous report was insufficient to induce osteocyte apoptosis.

In our transgenic mice, overexpression of *BCL2* in osteoblasts led to a reduction in the number of osteocyte processes. This suggests that Bcl2 alters cytoskeletal organization. Recently, it was reported that Bcl2 is able to form a complex with actin and gelsolin, which functions to decrease gelsolin-severing activity to increase actin polymerization, and to suppress cell adhesion, spreading, and motility [41]. Further, Bcl2 is an independent indicator of a favorable prognosis for all types of early-stage breast cancer [42], [43]. Thus, the reduction in osteocyte processes in *BCL2* transgenic mice may show the function of Bcl2, which modulates cytoskeletal reorganization.

The reduction in osteocyte processes in *BCL2* transgenic mice would result in a limited supply of oxygen, nutrients, and survival factors to osteocytes. In accordance with this hypothesis, Hif1-α protein, which is stabilized in hypoxia [44], and p53 protein, which is stabilized by hypoxia, nutrient deprivation, and withdrawal of survival factors in addition to DNA damage [45], [46], were markedly increased, and their target genes, including *Bax* and *Noxa*, were upregulated in osteocytes of *BCL2* transgenic mice. The expression of Mcl-1, which is a key member of the Bcl2 family of pro-survival proteins and undergoes proteasomal-dependent degradation during anoxia and growth factor withdrawal, was reduced in osteocytes [47], [48]. Further, Bim, which is upregulated by FOXO after the withdrawal of growth factors [49], and a truncated form of Bid (t-Bid), which is a product of caspase-8 mediated cleavage of Bid through death receptor signaling [17], increased. The failure to rescue osteocyte apoptosis by *p53* deletion also indicates that multiple signaling pathways were involved in osteocyte apoptosis. These findings strongly suggest that osteocyte apoptosis in *BCL2* transgenic mice was caused by an insufficient supply of oxygen, nutrients, and survival factors, probably due to the reduction of osteocyte processes.

Our *BCL2* transgenic mouse with accumulated dead osteocytes is a useful model to analyze the function of osteocytes because a repair process, which replaces dead osteocytes with new osteocytes by bone resorption and formation, was not evident in the mice irrespective of the massive accumulation of dead osteocytes. The functions of osteocytes in a physiological condition and an unloaded condition were examined using these mice (submitted to another journal). The gradual accumulation of death osteocytes and the reduction in the number of osteocyte processes, which will restrict the secretion of inflammation-inducible molecules to bone marrow, may have limited the repair reaction. We also successfully identified using *BCL2* transgenic mice that pyruvate dehydrogenase 4 (Pdk4) is one of the molecules responsible for bone loss in unloading [50].

In conclusion, osteoblast apoptosis is in part responsible for osteoporosis in sex steroid deficiency, glucocorticoid excess and aging, however, BCL2 inhibited osteoblast differentiation, impaired osteoblast function, and reduced the number of osteocyte processes. Thus, our results improve the comprehension of cellular mechanisms involved in osteoporosis.

## Supporting Information

Figure S1 **pQCT analysis.** The cortical bone density in 15 equal cross-divisions from metaphyses to diaphyses of femurs was measured in male wild-type mice (blue circles), tg(L) (red triangles), and tg(H) (yellow circles) of 10 weeks of age. Data are presented as the mean ± SD of 6 mice. *vs. wild-type mice, **p<0.01. pQCT analysis was performed using an XCT Research SA (Stratec Medizintechnick). The mineral density in cortical bone was analyzed using the threshold value, 690 mg/cm^3^.
(TIF)

Figure S2 **Northern blot and in situ hybridization analyses.** (A) Northern blot analysis of the bone matrix protein genes including *Col1a1*, *osteopontin*, and *osteocalcin*. RNA was extracted from the femurs of two male *BCL2* transgenic mouse lines (tg(L) and tg(H)) and wild-type mice (wt) at 4 weeks of age. Twenty micrograms of RNA was loaded and *Gapdh* was used as an internal control. (B–J) The expression of *Col1a1* (B–D), *osteopontin* (E–G), and *osteocalcin* (H–J) in male wild-type mice (B, E, H), tg(L) (C, F, I), and tg(H) (D, G, J) was examined by in situ hybridization at 8 weeks of age. Serial sections from tibiae were used for in situ hybridization and counterstained with methyl green. Scale bars: 0.5 mm.
(TIF)

Figure S3 **Histological analysis of** ***BCL2*** **transgenic mice at 1 week of age.** Sections from femurs of wild-type mice (A), tg(L) (B), and tg(H) (C) were stained with H-E, and the cortical bones are shown. Arrows in C indicate the lacunae containing cell debris. Scale bars = 0.1 mm.
(TIF)

Table S1 **Primer sequences.**
(XLS)
