# Supplementary figures and images for: Overexpression of Bcl2 in Osteoblasts Inhibits Osteoblast Differentiation and Induces Osteocyte Apoptosis
Source: PLoS One. 2011 Nov 17;6(11):e27487. doi: 10.1371/journal.pone.0027487 (PMC3219663; doi:10.1371/journal.pone.0027487)

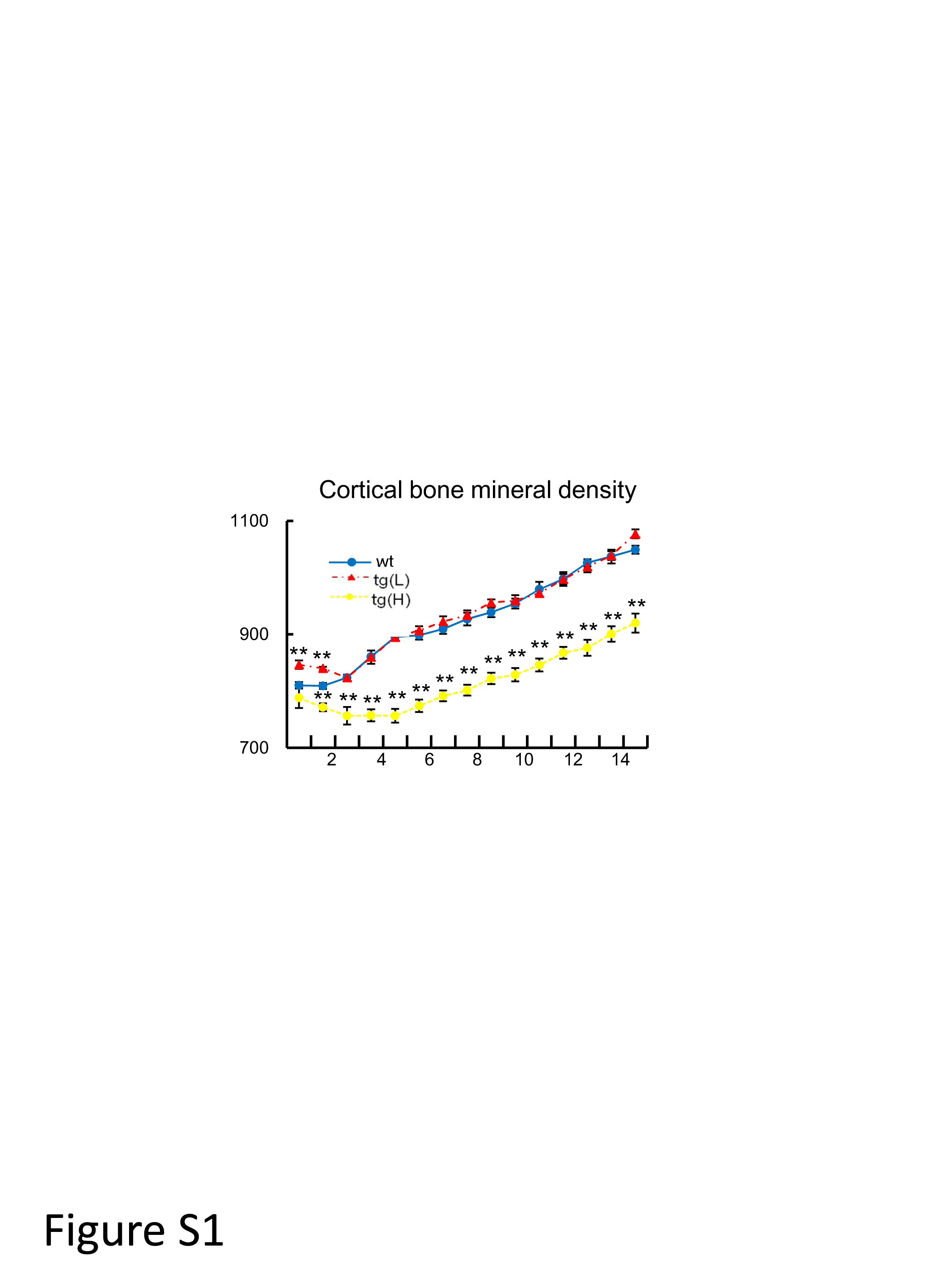

Supplement: Figure S1 — pQCT analysis. The cortical bone density in 15 equal cross-divisions from metaphyses to diaphyses of femurs was measured in male wild-type mice (blue circles), tg(L) (red triangles), and tg(H) (yellow circles) of 10 weeks of age. Data are presented as the mean ± SD of 6 mice. *vs. wild-type mice, **p<0.01. pQCT analysis was performed using an XCT Research SA (Stratec Medizintechnick). The mineral density in cortical bone was analyzed using the threshold value, 690 mg/cm3. (TIF) [file pone.0027487.s001.tif]

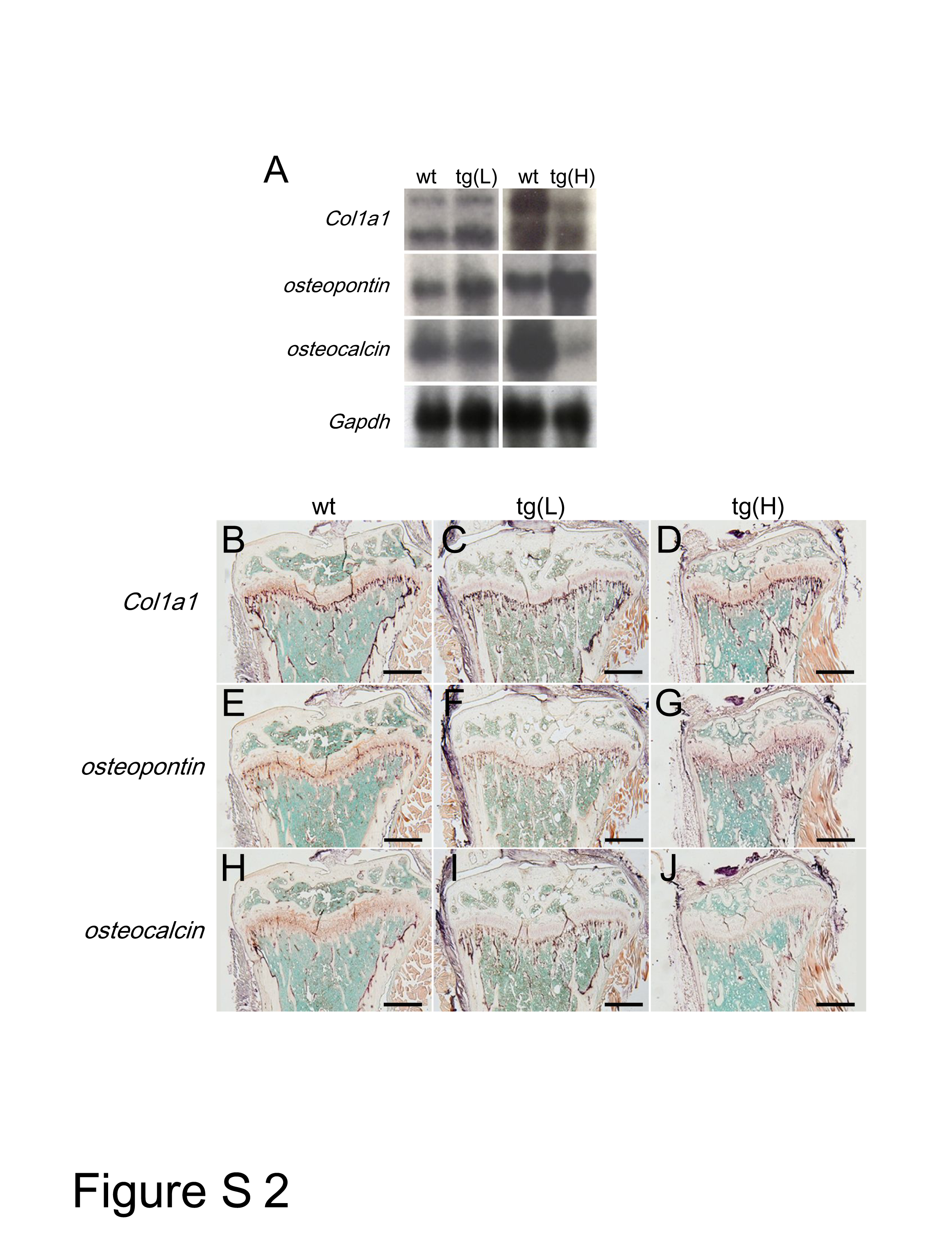

Supplement: Figure S2 — Northern blot and in situ hybridization analyses. (A) Northern blot analysis of the bone matrix protein genes including Col1a1, osteopontin, and osteocalcin. RNA was extracted from the femurs of two male BCL2 transgenic mouse lines (tg(L) and tg(H)) and wild-type mice (wt) at 4 weeks of age. Twenty micrograms of RNA was loaded and Gapdh was used as an internal control. (B–J) The expression of Col1a1 (B–D), osteopontin (E–G), and osteocalcin (H–J) in male wild-type mice (B, E, H), tg(L) (C, F, I), and tg(H) (D, G, J) was examined by in situ hybridization at 8 weeks of age. Serial sections from tibiae were used for in situ hybridization and counterstained with methyl green. Scale bars: 0.5 mm. (TIF) [file pone.0027487.s002.tif]

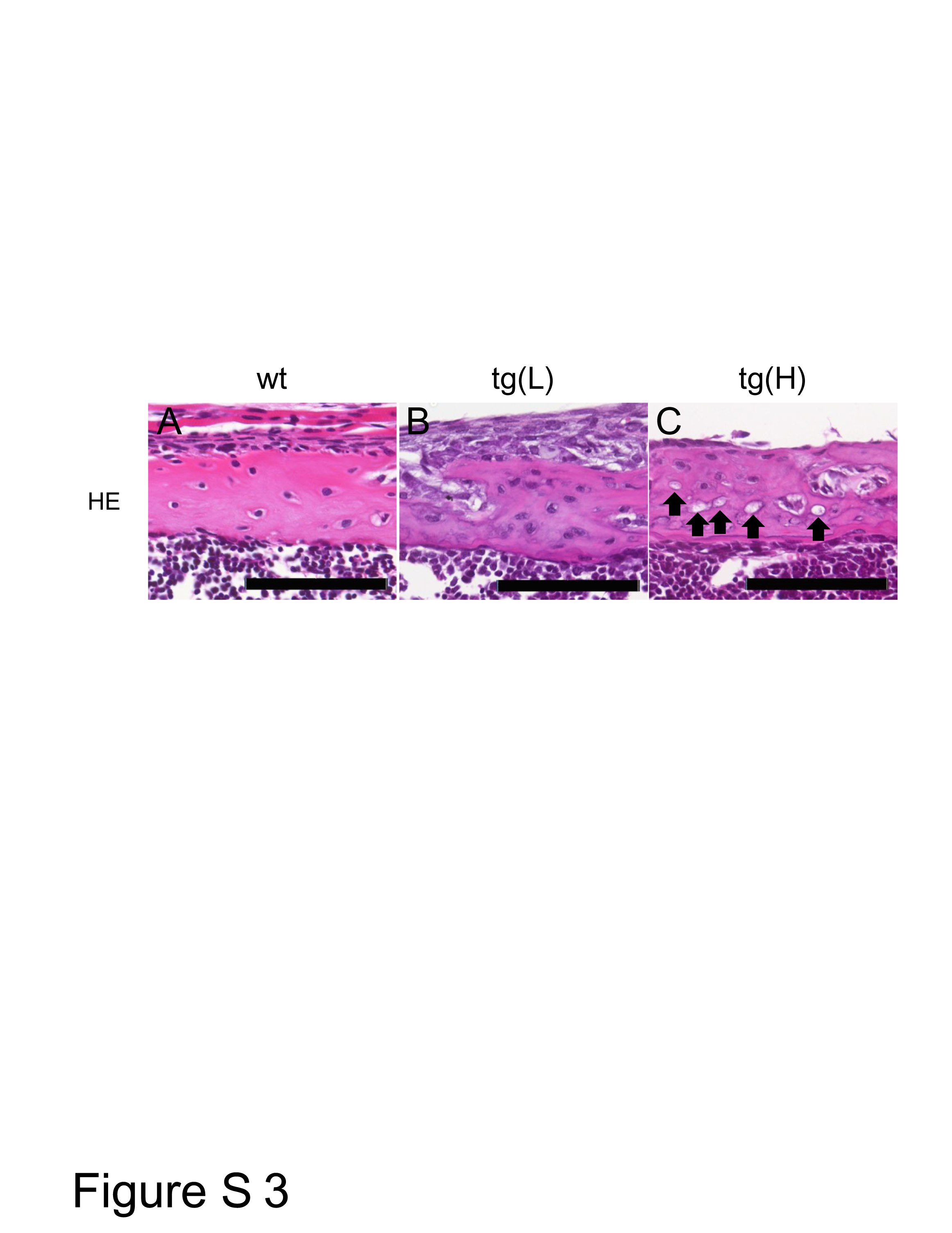

Supplement: Figure S3 — Histological analysis of BCL2 transgenic mice at 1 week of age. Sections from femurs of wild-type mice (A), tg(L) (B), and tg(H) (C) were stained with H-E, and the cortical bones are shown. Arrows in C indicate the lacunae containing cell debris. Scale bars = 0.1 mm. (TIF) [file pone.0027487.s003.tif]
